# Non-Enzymatic Phenylboronic Acid-Based Optode Membrane for Glucose Monitoring in Serums of Diabetic Patients and in the Culture Medium of Human Embryos

**DOI:** 10.3390/s22197135

**Published:** 2022-09-21

**Authors:** Mohamed M. Taha, Mahmoud S. Rizk, Mohamed A. Zayed, Fatehy M. Abdel-Haleem, Ahmed Barhoum

**Affiliations:** 1 Chemistry Department, Faculty of Science, Cairo University, Giza 12613, Egypt; 2 Adam International Hospital, Aden Street Mohandesein Anas Ibn Malek, Giza 12411, Egypt; 3 Center for Hazards Mitigation, Environmental Studies and Research (CHMESR), Cairo University, Giza 11795, Egypt; 4 NanoStruc Research Group, Chemistry Department, Faculty of Science, Helwan University, Cairo 11795, Egypt; 5 National Centre for Sensor Research, School of Chemical Sciences, Dublin City University, D09 V209 Dublin, Ireland

**Keywords:** glucose sensor, optochemical sensor, ionophore, chromoionophore, detection mechanism, ion-selective membrane, plasticizers

## Abstract

Monitoring glucose levels is important not only for diabetics, but also for tracking embryonic development in human embryo culture media. In this study, an optochemical sensor (glucose-selective polymer membrane) was fabricated for the determination of glucose in serum from diabetic patients and the culture media of human embryos. The optode membranes were formulated using polyvinyl chloride (PVC) as the polymer matrix and 4′,5′-dibromofluorescein octadecyl ester (ETH 7075) as the chromoionophore. The sensitivity of the optode membranes was optimized using two different plasticizers (tricresyl phosphate-TCP and nitrophenyloctyl ether-NOPE) and three ionophores (nitrophenylboronic acid-NPBA, trifluorophenyboronic acid-TFPBA, 4′-nitrobenzo-15-crown-5) and tested for glucose detection. The best optode membrane was formulated from 49.5% PVC, 49.5% TCP, 1% NPBA, and 1% ETH 7075. It showed a linear dynamic range of 10^−3^ M to 10^−1^ M, with a detection limit of 9 × 10^−4^ M and a response time of 2 min. The detection mechanism involves H-bonding between NPBA and glucose, which was confirmed by Fourier transform infrared (FTIR) and nuclear magnetic resonance (NMR). The reaction also involves the formation of boronate esters in basic media with deprotonation of the chromoionophore (ETH 7075), leading to a decrease in UV–Vis absorbance at λmax = 530 nm. The membrane optode was used for glucose determination in synthetic culture medium, commercial embryo culture medium (GLOBAL^®^ TOTAL^®^ W/HEPES), and serum from normal and diabetic patients, showing good accuracy and precision of the optode.

## 1. Introduction

According to the World Health Organization (WHO), about 422 million people have diabetes, and 1.6 million die each year as a result of diabetes [1]. Glucose monitoring can be used as an indicator of diabetes; normal glucose concentration is between 70 mg/dL and 100 mg/dL (from 3.9 to 5.6 mM) [2,3]. Controlling glucose concentration in the media used for embryo culture is an important factor for the optimal development of embryos in vitro [4,5]. The commercially available culture medium (GLOBAL^®^ TOTAL^®^ W/HEPES) from LifeGlobal^®^ is intended for the culture of human embryos from zygote to blastocyst and embryo transfer. During in vitro culture of human embryos with the onset of embryonic genome activation, a switch to glucose as a nutrient occurs [6]. It was confirmed that glucose consumption on day 4 was higher in embryos that developed than in embryos that did not develop [7,8]. Rapid screening of glucose metabolism of the human embryo on days 4 and 5 may prove to be a useful benchmark for developing algorithms to select embryos to be transferred to humans for in vitro fertilization [9].

Since it is important to control glucose in this medium, several techniques have been described for this purpose. Glucose uptake was first studied spectrophotometrically in bovine blastocysts on day 10 [10]. Since the diameter of the bovine embryo at this time is approximately 10 times larger than that of the human embryo, glucose uptake from the culture medium could be measured spectrophotometrically. In a follow-up study, ultra-microfluorescence was used to analyze glucose uptake by individual mouse blastocysts before uterine transfer; embryos that implanted and developed into a fetus had a significantly higher rate of glucose consumption in vitro than those that did not [11]. The analytical method was a miniaturized version of conventional enzymatic analytical methods. Instead of cuvettes, the reactions were performed in 50 µL drops on siliconized slides, and the reaction products were quantified using a fluorescence microscope with photomultiplier tubes (PMT), which made this method expensive [11,12]. Glucose biosensors are usually based on the two enzyme families, glucose oxidase enzyme (GOx) and Glutamate dehydrogenase (GDH). The immobilized GOx enzyme catalyzes the oxidation of β-D-glucose by molecular oxygen producing gluconic acid and H_2_O_2_. Then, the H_2_O_2_ is determined optically or electrochemically.

Optode sensors have been developed to detect a wide range of cations, anions, neutral species, and glucose [13,14,15,16,17]. Previous studies used the enzyme glucose oxidase, quantum dots, polyaniline, and near-infrared dyes [18,19,20,21,22,23,24]; these sensors have a detection limit of 10^−3^ M and usually require the use of glucose oxidase enzymes, which are expensive and require sample preparation with a long incubation time [18,19,20,21,22,23,24]. To overcome these problems, we report the first optode membrane sensor based on pH chromoionophores and phenylboronic acid as ionophores for glucose detection, without using a glucose oxidase enzyme. The low cost, increased selectivity, pH cross-sensitivity, micromolar detection limit, incubation time, and internal solution not required made the developed optode very advantageous. The use of a separate chromoionophore simplified the mechanism of signal transmission between the detection of the analyte and the corresponding output. The sensor showed high selectivity for glucose over other interfering species in the culture medium, i.e., sodium pyruvate, arginine, glutamic acid, tryptophan, and leucine. 

## 2. Experimental

### 2.1. Materials 

All chemicals were of analytical grade. 3-nitrophenylboronic acid (NPBA, 99%), 3,4,5-trifluorophenyboronic acid (TFPBA, 95%), 4-nitrobenzo-15-crown-5 (crown-5, 99%), 4,5-dibromofluorecein octadecyl ester (ETH 7075, 99%), polyvinyl chloride (PVC), nitrophenyl octyl ether (NOPE, 99%), tricresyl phosphate (TCP, 98%), deuterated dimethyl sulfoxide (DMSO, 99%) (in which hydrogen is replaced by deuterium), and tetrahydrofuran (THF, 98%) were purchased from Sigma-Aldrich (Schnelldorf, Germany) and used to prepare PVC optode membrane. Anhydrous glucose (C_6_H_12_O_6_, 99%), sodium hydroxide (NaOH, 99%), glycine (C_2_H_5_NO_2_, 99%), and sodium acetate trihydrate (C_2_H_9_NaO_5_, 96%) were purchased from El Nasr Pharmaceutical Chemicals (Egypt). Phosphoric acid (H_3_PO_4_, 85%), disodium hydrogen phosphate (Na_2_HPO_4_, 99%), 2-amino-2- (hydroxymethyl)propane-1,3-diol (Tris, 99%), sodium pyruvate (C_3_H_3_NaO_3_, 99%), L-glutamic acid (C_5_H_9_NO_4_, 99%), sodium lactate (NaC_3_H_5_O3, 60% solution), L-arginine (C6H_14_N_4_O_2_, 99%), L-phenylalanine (C_9_H_11_NO_2_, 99%), L-tryptophan (C_11_H_12_N_2_O_2_, 99.5%), L-leucine (C_6_H_13_NO_2_, 98%), and glacial acetic acid (CH_3_COOH, 99%) were purchased from Loba Chemie (Maharashtra, India) and used in the experiments for preparation calibration curves, selectivity test, and all other measurements.

### 2.2. Solution Preparation 

Phosphate buffer (0.05 M Na_2_HPO_4,_ pH = 8.0), Tris buffer (0.02 M Tris, pH = 9), and glycine/NaOH buffer (0.02 M glycine, pH = 10.5) were prepared by titrating the solutions with H_3_PO_4_, HCl, and NaOH, respectively, to the desired pH values [25,26,27]. The glucose stock solution (10^−1^ M) was prepared by dissolving the appropriate amount of glucose in 100 mL of buffer solution, and the diluted solutions (between 10^−1^ M and 10^−4^ M) were prepared from the appropriate dilutions. A synthetic mixture of the culture medium of the same ingredients as the commercially embryonic culture medium was prepared as described in the literature [3]: 2.0 × 10^−3^ M glucose, 4.8 × 10^−3^ M sodium lactate, 2.0 × 10^−4^ M sodium pyruvate, 3.0 × 10^−4^ M arginine, 0.5 × 10^−4^ M L-glutamic acid, 2.8 × 10^−5^ M L-tryptophan, 2.3 × 10^−4^ M L-leucine, and 1.1 10^−4^ M L-phenylalanine. Commercial human embryo culture medium (GLOBAL^®^ TOTAL^®^ W/HEPES) was used as glucose-rich samples for real applications. The medium and solutions were stored in the refrigerator for protection.

### 2.3. Instrumentations

A UV–Vis spectrophotometer (UV–Vis OPTIZEN POP, Daejeon, Korea) was used to record various spectra of the prepared optodes. High-performance liquid chromatography (HPLC, YL9100, Anyang, Korea) was used for the detection of glucose in a commercial human embryo culture medium (GLOBAL^®^ TOTAL^®^ W/HEPES). pH meter (The pH meter (Adwa AD1030, Szeged, Hungary)) was used to measure solutions. Fourier transform infrared spectroscopy (FTIR, SHIMADZU IR, Tokyo, Japan) and nuclear magnetic resonance spectroscopy (NMR, Varian Mercury VX-300 NMR, USA) were used to study the chemical bonds in the glucose, ionophore (NPBA), and their complex. The atomic force microscope (AFM, 5600LS Agilent, Schwalmstadt, Germany) was used to characterize the surface roughness (morphology) of the optode membranes.

### 2.4. Optode Preparation

Optical sensing membranes were prepared manually using a micropipette. The membrane cocktail was prepared by dissolving the various optode components in 2 mL THF, and the cocktail amount was poured onto a dust-free 0.9 × 4 cm^2^ glass slide with a thickness of 1 mm; then, the membrane was allowed to air dry for about 20 min to form the optode and stored in the dark when not in use. The resulting films had a thickness in the range of 4–9 μm [15]. Absorbance measurements were performed by placing the optode in a plastic cuvette (1 × 1 × 4 cm^3^) containing a glucose solution of different concentrations. The optode was conditioned in the selected buffer for 20 min before the first measurement. The buffer solution was used as a blank to correct for background absorbance. The pH measurements were performed using a pH meter.

### 2.5. Selectivity

One of the most important properties of optodes is their selectivity. The real embryo media, GLOBAL^®^ TOTAL^®^ W/HEPES, contains amino acids, such as alanine, aspartic acid, asparagine, arginine, cysteine, glutamic acid, glycine, histidine, isoleucine, leucine, lysine, methionine, phenylalanine, proline, serine, threonine, tryptophan, tyrosine, and valine, and other salt ions of calcium, phosphorus, potassium, chloride, sodium, magnesium, aluminum, chromium, cobalt, and manganese; it contains also sugars, such as glucose, lactate, and pyruvate [3]. Briefly, 100 mL of solution (10^−1^ M glucose and 10^−2^ M of the interfering ion) was prepared in glycine/NaOH buffer (pH 10.5). The interfering species (sodium pyruvate, L-arginine, L-glutamic acid, L-tryptophan, sodium bicarbonate, potassium dihydrogen phosphate, L-leucine, sodium lactate, and L-phenylalanine) were selected for the study because they are present in higher concentrations in the culture medium and can react with boronic acid via hydrogen bonds [17,28,29]. The optical response of different glucose solutions (10^−1^ M) was recorded in the absence and presence of 10^−2^ M interfering species.

### 2.6. Effect of Soaking Time and Regeneration 

Soaking time is the time between the insertion of the optode into the test solution and the first measurement [30]; the effect of soaking time was studied by soaking the membrane in the buffer for 5 min, 20 min, and 1 h and recording the absorbance spectrum of glucose solutions (from 10^−4^ to 10^−1^ M) at each time to select the best soaking time. Regeneration of the optode means that the electrode is immersed in buffer or acid to restore its equilibrium state before the last measurements.

### 2.7. Response, Reversibility, and Lifetimes 

The response time is the time required to reach 95% of the equilibrium response [30]. It was measured by continuously measuring the optode membrane absorbance in the different buffered glucose concentrations (0.02 M glycine/NaOH buffer, pH 10.5) at λ_max_ = 530 nm every 30 s until it reached a constant value in three measurements. Reversibility means that the different concentrations are measured from low to high concentrations, followed by measurements in the reverse direction [31]. Reversibility was examined for the best optode by measuring the UV–vis absorbance at λ_max_ = 530 nm after soaking the optode forward and backward between two different glucose concentrations (between 10^−1^ M and 10^−3^ M). The lifetime was measured by taking measurements with the best optode membrane to obtain the calibration curve, and the change in liner dynamic concentration range and limit of detection were recorded. Optode lifetime was determined by plotting a calibration curve for glucose in the concentration range of 10^−4^ to 10^−1^ M over the day to determine the time after which the membrane could no longer be used for measurements.

### 2.8. Repeatability and Reproducibility 

Repeatability is the measurement accuracy of the same analyst using the same equipment and solutions in a single laboratory period. If one of the measurement conditions changes, reproducibility is the precision [31,32]. Repeatability is represented by the display of error bars in the calibration curve, where reproducibility was checked by monitoring sensor-to-sensor deviation.

### 2.9. Analytical Application

Glucose provides the reducing power needed to neutralize oxidative species (oxidative stress) that form in vivo and in vitro. The analytical benefit of the developed optode membrane was tested by the determination of glucose in a synthetic mixture that has the same composition as that of the real media. The optode was used to also monitor glucose in the real human embryos’ culture medium and the results were compared with that obtained by the reference method previously reported in the literature [3]. For determination of glucose in diabetes serum, 0.5 mL of the serum was diluted to 10 mL using buffer, and the solution was measured by the optode and by using a reference kit (GACO20, Sigma Aldrich) that depends on enzymatic measurements followed by spectrophotometric determination at 540 nm [2]. The recovery and the standard deviation were calculated to confirm the reliability and precision of measurements, respectively.

## 3. Results and Discussion

Optode membrane sensor developed in this study provides an alternative, minimally invasive tool for monitoring glucose levels, for example, in culture media and diabetes research. The optode membrane was used for glucose determination in synthetic medium, commercial true embryo medium (GLOBAL^®^ TOTAL^®^ W/HEPES), and serum from diabetic patients, showing good accuracy and precision of the optode. Extension of the glucose optode membrane will enable improved monitoring of other physiological processes.

### 3.1. Effect of Membrane Composition

**Figure 1:**
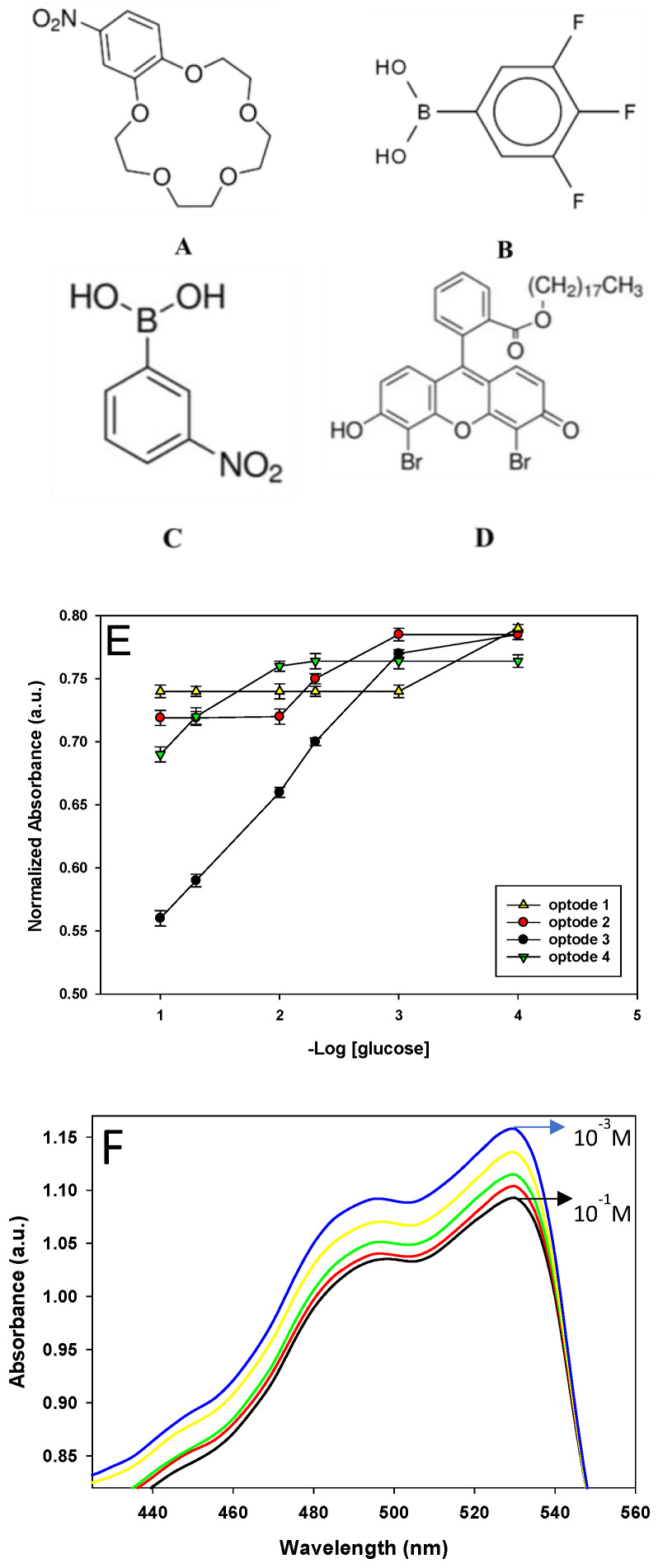
Chemical structures of the three ionophore (**A**) 4-nitrobenzo-15-crown-5, (**B**) 3,4,5-trifluorophenylboronic acid, (**C**) 3-nitropheny boronic acid; and (**D**) ETH 7075 chromoionophore. (**E**) Calibration curves of different optodes measured at λmax = 530 nm, the standard deviation was obtained through 5 measurements (**F**) UV-vis spectra of optode 3 at different concentrations increased gradually from 10^−3^ to 10^−1^ M.

Membrane composition is the most important factor affecting sensitivity, detection limit, selectivity, and other properties [31]. The membranes were prepared from PVC as the polymer matrix and ETH 7075 as the chromoionophore. PVC is the third most produced synthetic plastic in the world, with an annual production of about 40 million tons. It has also been extensively studied as a plastic membrane for the production of optode sensors. However, its use requires a plasticizer to improve the diffusion mobility of the analyte to bind with the ionophore. The chromoionophore VI (ETH 7075) is a lipophilic pH indicator and is generally used in the preparation of optical sensors. ETH 7075 was used to prepare an optical membrane used for the determination of glucose. The sensitivity of the membrane for glucose detection was optimized using two different plasticizers (TCP and NOPE) and three ionophores (NPBA, TFPBA, and Crown-5) (see Table 1 and Figure 1). The hydrogen bonding and the selective interaction between the ionophore and analyte is key for developing the membrane optodes [31], especially for glucose using phenyl boronic acid as an ionophore [32,33,34,35,36,37]. Optode 1 fabricated with Crown-5 (ionophore 2) and TCP (plasticizer 1) exhibits a linear dynamic range of 10^−4^ M to 10^−3^ M, with a detection limit of 10^−4^ M. Optode 2 fabricated with TFPBA (ionophore 2) exhibits a linear dynamic range of 10^−3^ M to 10^−2^ M, with a detection limit of 10^−3^ M. Optode 3 (ionophore 3) fabricated from NPBA exhibits a broader linear dynamic range of 10^−3^ M to 10^−1^ M, with a detection limit of 9 × 10^−4^ M. Optode 4 fabricated from NPBA (ionophore 3) but with NPOE as plasticizer exhibits a smaller linear dynamic range of 10^−2^ M to 10^−1^ M, with a detection limit of 1 × 10^−2^ M. 

Crown 5 (ionophore 1) forms a weaker H-bond with glucose compared to the other ionophores (NPBA and TFPBA), Figure 1. This can be explained by the large cavity of Crown 5, which is not well fitted for the glucose molecule, and by weak hydrogen bonds in interactions [22,38]. Ionophores 2 and 3 are boronic acid derivatives (TFPBA and NPBA) that have different electron-withdrawing groups (three fluorides and one nitro group, respectively). These ionophores (TFPBA and NPBA) show different strengths of H-bonding with glucose [35,36,37]. The response characteristics of optodes 2 and 3 show that NPBA (ionophore 3) has a higher affinity for glucose than TFPBA (ionophore 2); see Table 1 and Figure 1F. The TFPBA (ionophore 2) contains three fluoride groups with very high electronegativity. The inductive effect of these groups is distributed over three positions (metal and para) on the benzene ring (Figure 1B), which attenuates their electron-withdrawing effect. In addition, the three fluorides cause steric hindrances that can delay the approach of glucose to hydrogen bonding with TFPBA. The NPBA (ionophore 3) contains a nitro group in meta-position (Figure 1D) with a very high electronegativity and electron-withdrawing effect than the three fluoride groups of TFPBA (Figure 1B). Accordingly, the NPBA ionophore exhibited the highest interaction probability than TFPBA, which was further confirmed by the dynamic decrease in absorbance at λmax = 530 of optode 3 which incorporates NPBA (see Figure 1F).

**Figure 2:**
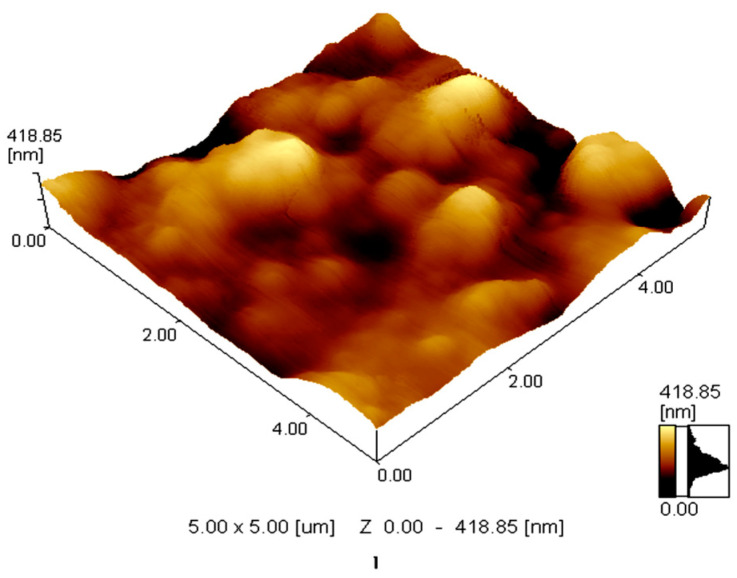
AFM image of the prepared membrane of the Optode 4 based on NOPE as a plasticizer.

Previous studies have shown that the selectivity and sensitivity of PVC membranes can be affected by the polarity of the plasticizer [31]. Plasticizer molecules can diffuse into the PVC matrix, reducing the interactive forces and allowing homogeneous distribution of ionophores and chromophores in the membrane. Plasticizers of different polarities lead to different effects due to the interactions between PVC polymer, ionophore, chromophore, and analyte. Thus, the effects of plasticizer type on optodes sensitivity were studied. As shown in Figure 1E, Optode 4 made with NOPE as plasticizer exhibited a lower linear concentration range (10^−2^ M to 10^−1^ M) compared to Optode 3 (10^−3^ M to 10^−1^ M). The NOPE has limited solubility for the membrane cocktail compared to TCP (Optode 3). These results were confirmed by the AFM images shown in Figure 2. The presence of peak areas with brighter colors indicates the insoluble part of the membrane of Optode 4. Thus, the inhomogeneity of optode 4 reduces the sensitivity and reproducibility of the glucose detection compared to Optode 3.

### 3.2. Recognition Mechanism 

Commercially available glucose sensors are based on the catalytic action of the enzyme glucose oxidase and optical or electrochemical detection of the reaction by-products, such as (H_2_O_2_) [16]. Optode 3 membrane contains pH chromoionophores (ETH7075) and ionophores (NPBA) to detect glucose without using glucose oxidase enzyme. Recognition of glucose by the NPBA results in a decrease in the pKa value of the NPBA. This decrease in pKa value leads to increased acidity of the solution, which is monitored by ETH7075 (pH-sensitive dye) [29,30]. NPBA (Lewis acid) can interact with OH ions in the (buffered) sample solution, resulting in a conformational change in the boron atom from sp2 to sp3 [17]. The pH of the solution reaches the NPBA pKa (8.9) when the concentration of NPBA (acid form) is equal to the concentration of their boronate form [17]. However, the addition of glucose (diols) to the solution shifts the equilibrium and leads to a decrease in the apparent pKa value Equation (1) [18]. The NPBA boronate form binds the OH groups (diols) of glucose molecules and forms a cyclic ester, leading to consumption of the boronate form. To restore the acid–base balance, the NPBA acid form reacts with more OH ions to form more boronate. Consequently, the higher concentration of glucose (diols) leads to a decrease in the NPBA acid form and, thus, to a decrease in the apparent pKa value. As shown in Equation 2, as the glucose concentration increases, the number of protons released into the membrane during the reaction increases. As a result, the pH of the membrane decreases and, consequently, the pH of the test sample also decreases.

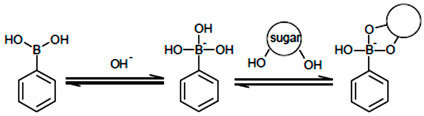
(1)

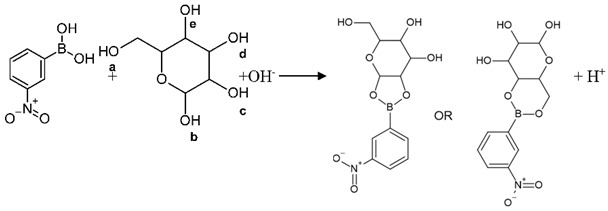
(2)

H^+^ + Ind^−^ = IndH (3)

**Figure 3:**
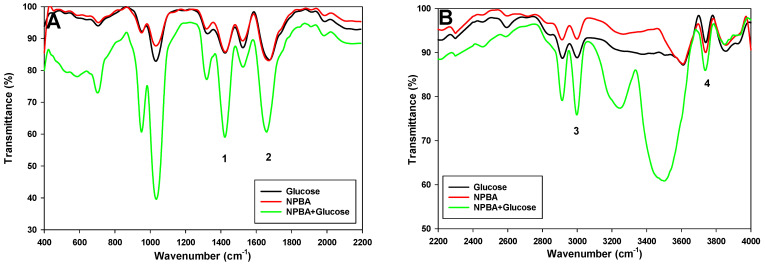
IR spectra of glucose, NPBA, and their mixture (complex): (**A**) IR spectra from 400 to 2200 cm^−1^; (**B**) IR spectrum from 2200 to 4000 cm^−1^.

NPBA can selectively interact with carbohydrate sugars, especially glucose, and this interaction can be transferred with the pH indicator dye ETH 7075, which causes a signal change when glucose is bound (Equation (3)). This increases the amount of chromoionophore in the protonated form, leading to a decrease in absorbance at λmax = 530 nm, as seen in the calibration curves in Figure 1 [15]. This mechanism was confirmed by following the change in solution pH with time for the different concentrations, FTIR, and ^1^H-NMR of glucose and NPBA before and after binding. As can be seen from the FTIR spectra in Figure 3, the broad, strong band at 3741 cm^−1^ (assigned to band 4 in Figure 3) corresponding to the OH group of glucose was shifted to 3734 cm^−1^ due to intermolecular hydrogen bonding; the broadening of the peak is due to the presence of multiple OH in the glucose molecule. The two bands at 3032 cm^−1^ and 2939 cm^−1^ (assigned to band 3 in Figure 3), corresponding to the CH stretching in glucose, were shifted to 3001 cm^−1^ and 2916 cm^−1^ after interaction with the ionophore, confirming the intermolecular hydrogen bonding between these molecules. The band at 1674 cm^−1^ and 1427 cm^−1^ (assigned 2 and 1 in Figure 3) in glucose, corresponding to C = O and CH bending, respectively, was shifted to 1658 cm^−1^ and 1416 cm^−1^ [38,39]; this decrease in wavenumber is due to the intermolecular hydrogen bonding between glucose and the ionophore. 

^1^H-NMR confirms the interaction between the glucose and the ionophore NPBA by changing the main peaks of the spectrum with the change in the chemical shift to lower values, as shown in Figure S1 (Supplementary Materials) and listed in Table 2 [40]. FTIR and ^1^H-NMR were performed in solid state and deuterated DMSO, respectively; this facilitates the presence of hydrogen bonding, followed by a chemical reaction in the presence of the OH ions in the analyte solution to form an ester product Equation (2). As shown in Equation (2), glucose can react with the two OH groups (a and e) or the other two hydroxides (c and b). This was confirmed by the change in the chemical shift for each of these hydroxides, as shown in Table 2.

### 3.3. Effect of pH 

**Figure 4:**
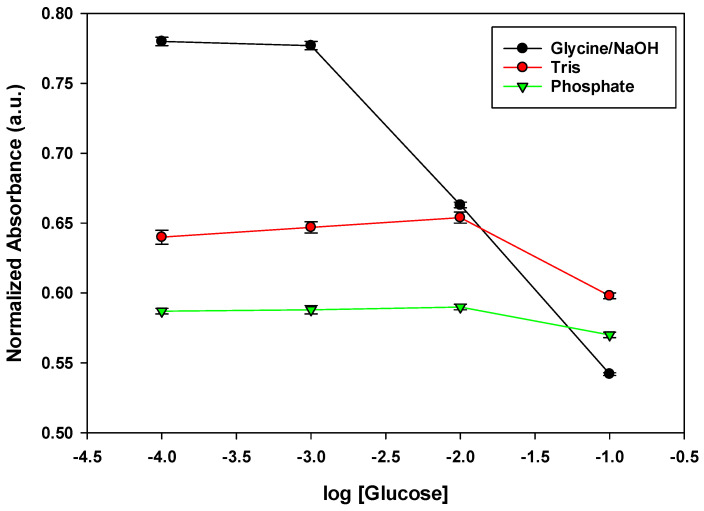
Calibration curves with error bars for optode 3 in different pH and buffers. The standard deviation was obtained through 5 measurements.

The pH cross-sensitivity of optodes is well known and means that the linear dynamic concentration range changes by changing the pH of the measured solutions [41]; pH control is an important condition in glucose determination. It is known that phenylboronic acid derivatives can bind diol compounds, including sugars, to form cyclic boronate esters that are negatively charged by the addition of OH ions from solution [42]. Glycine/NaOH buffer (pH = 10.5) showed the greatest decrease in absorbance at a glucose concentration of 10^−1^–10^−3^ M at λ_max_ = 530 (Figure 4). For Tris buffer (pH = 9.0) and phosphate buffer (pH = 8.0), absorbance dropped to a very low value in a narrower concentration range of 10^−1^ M to 10^−3^ M at λmax = 530 (Figure 4). The complexation equilibrium between several model diols and monosaccharides was determined by the method of pH depression [43]; it was found that the ester formation was more favorable in high pH solutions where the boronate ion was present in high concentrations (Figure 4) [43]; this explains the largest decrease in absorbance in the case of glycine/NaOH buffer and the smallest decrease in the case of phosphate buffer.

### 3.4. Interference Study

**Figure 5:**
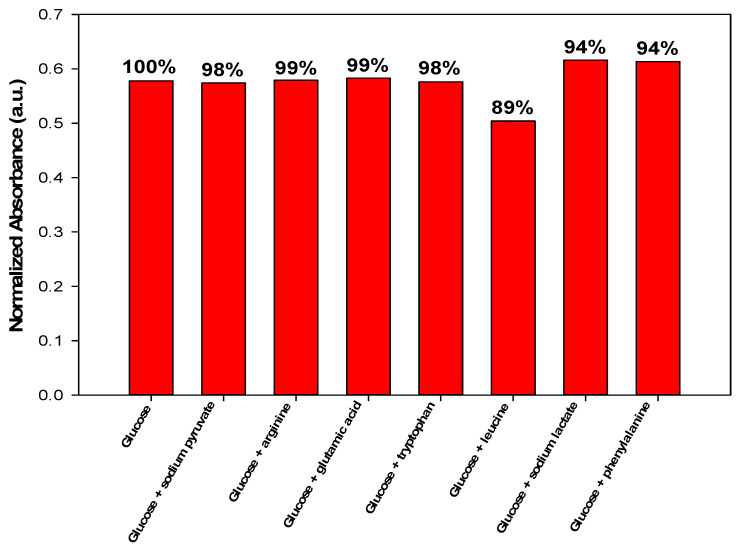
Effect of different interfering species on the response of optode 3 in presence of glucose.

The effect of interfering substances on the efficiency of glucose determination with Optode 3 was studied (10^−1^ glucose, 10^−1^ glucose along with 10^−2^ interfering substances), as given in Table S1 (Supplementary Materials) and Figure 5. The interfering substance was added to the glucose solution at a concentration one order of magnitude lower to match the composition of the embryo medium. As shown in Figure 5, the interfering substance caused a small change in absorbance, less than 7%, with a signal recovery of 93%. This confirms the selective interaction between the ionophore and glucose in the presence of other interfering species. Of all the interfering species, leucine, lactate, and phenylalanine showed the greatest change in absorbance. This can be explained by the ability of phenylboronic acid to interact with amino acids [44,45,46]. To further confirm the high selectivity of NPBA for glucose, the different interfering species were tested with glucose at different concentrations (Table S2, in Supplementary Materials). The results confirm the specificity of NPBA for glucose. Other ionic species (sodium, potassium, and ammonium) were not tested because NPBA has little tendency to combine with them. 

### 3.5. Response Time and Reversibility

**Figure 6:**
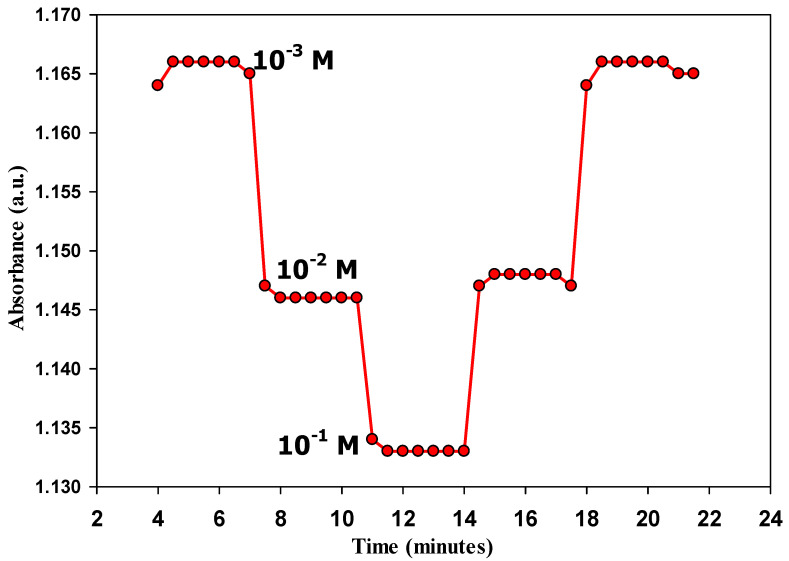
Reversibility and response time of optode 3 in response to different glucose solutions (10^−3^ M, 10^−2^ M, and 10^−1^ M).

The response time of optode 3 was followed by recording the absorbance at different times [15,31]. It was found that the soaking time at high (10^−1^ M) and low concentrations (10^−3^ M) was 1 and 2 min, respectively (Figure 6). This is consistent with the literature that lower concentrations require a longer time for a stable response [15,31]. Reversibility is related to kinetic constraints and exchange kinetics at the solution–membrane interface [31], i.e., diffusion rather than complex formation is the rate-determining step. Moreover, the change in ligand conformation and the lipophilicity of the membrane matrix are other reasons that control reversibility [31]. Optode 3 showed reliable reversibility (Figure 6); it recovered about 92% of its signal and the small degradation can be attributed to the presence of the diffusion layer during soaking, and this diffusion layer is the kinetically limiting factor [31].

### 3.6. Soaking, Regeneration, and Lifetime

Soaking is an important process for stabilizing the response of optodesensors [31]. A soaking time of about 20 min before first use is sufficient for membrane activation of optode 3 [12,47,48]. Regeneration was tested by soaking optode 3 in 0.1 M NaOH to deprotonate the chromoionophore ETH 7075. After soaking, the calibration curve was recorded, followed by soaking in buffer solution [49]. The regeneration step can only be performed with buffer solution but it takes several hours. However, a strong base shortens this time to a few minutes, which is sufficient to recover about ≥ 95% of the signal [49]. This time reduction is beneficial to minimize the dissolution of the optode in the measurement or soaking solutions and to shorten the measurement time so that it is suitable for routine analysis and reuse of the optode. After several minutes of immersion in the base, Optode 3 should be soaked in buffer solution for 5–10 min to remove the excess base. In terms of lifetime, it was found that Optode 3 can be used for one day, and the sensing membrane still works after this period but it is not bonded to the quartz plate of the optode. Other polymers, polyurethane, or the use of other substrates could be suitable to solve this problem [31].

### 3.7. Repeatability and Reproducibility

**Figure 7:**
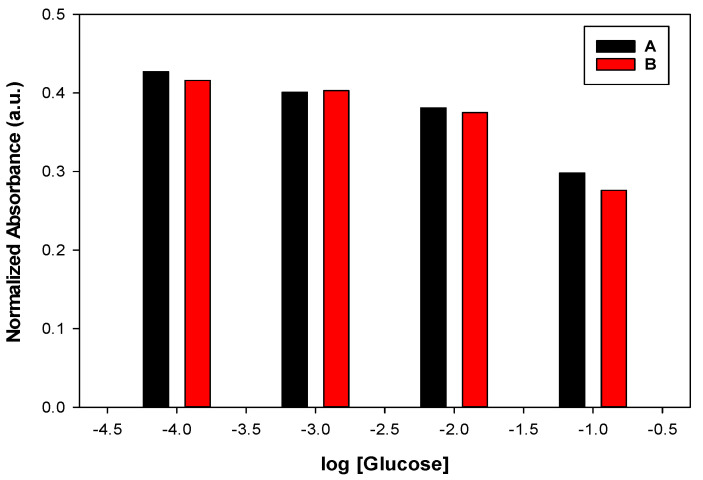
Reproducibility of the two optodes (A, B) prepared from the same composition of optode 3 in response to different glucose solutions (10^−4^ M, 10^−3^ M, 10^−2^ M, and 10^−1^ M).

Repeatability was shown as error bars in the calibration curves in Figure 1, where error bars were calculated for five measurements. It can be observed that the values of the error bars are very small, confirming the repeatability of the same sensor under the same conditions. The reproducibility was expressed using two different sensors (sensor-to-sensor variation) to show the sensor-to-sensor variation (Figure 7). The absolute values of the two sensors A and B are comparable, as shown in Figure 7.

### 3.8. Analytical Detection of Glucose in Real Samples 

Practical analytical usefulness of the developed optode 3 was tested by the determination of glucose in both synthetic culture medium and commercial culture medium of human embryos (GLOBAL^®^ TOTAL^®^ W/HEPES); the results were compared with the theoretical value or that of the reference method (HPLC) to calculate the recovery. As shown in Table 3, optode 3 was successfully used for the determination of glucose in synthetic culture media prepared to match the composition of the commercial embryo media (GLOBAL^®^ TOTAL^®^ W/HEPES), as confirmed by the recovery values. Optode 3 has also been successfully used for the determination of glucose in serum from normal and diabetic patients. Serum samples were determined using both Optode 3 and reference glucose kits based on enzymatic analysis (glucose assay kit GACO20, Sigma Aldrich), followed by spectrophotometric determination at λmax = 540 nm [2]. Optode 3 showed a high sensitivity for reliable determination of serum samples with a good recovery near the detection limit. In all applications, the sample was determined six times with a very low standard deviation, which was confirmed by repeatability and reproducibility measurements.

## 4. Conclusions

Novel optode membranes have been developed for glucose detection using a simple and inexpensive method that does not require glucose oxidase enzymes. The composition of the optode membranes was optimized using polyvinyl chloride (PVC) as the polymer matrix and 4′, 5′-dibromofluorescein octadecyl ester (ETH 7075) as the chromophore, three different types of ionophores (nitrophenylboronic acid-NPBA, trifluorophenyboronic acid-TFPBA, and 4′-nitrobenzo-15-Crown-5), and two different plasticizers (tricresyl phosphate-TCP and nitrophenyl octyl ether- NOPE). The best optode membrane was formulated using TCP as the plasticizer and NPBA as the ionophore, which exhibited a linear dynamic range of 10^−3^ M to 10^−1^ M, with a detection limit of 9 × 10^−4^ M and a response time of 2 min. The optode showed good accuracy and precision and was highly selective in glucose-rich synthetic medium, commercial true embryo medium (GLOBAL^®^ TOTAL^®^ W/HEPES), and serum from diabetic patients. The detection mechanism is based on H-bonding between the ionophore (NPBA) and glucose molecules, followed by the formation of a boronate ester, and then deprotonation of the chromophore dye (ETH 7075), which results in a decrease in UV–vis absorbance at 530 nm.

## Figures and Tables

**Table 1:** Composition of the prepared optode membranes and their response characteristics towards glucose.

Optodes	Optode Composition, (wt%)				Optode Response, M	
	PVC	Plasticizer	Ionophore	ETH7075	Conc. Range	LOD
Optode 1	49.0	49.0 (TCP)	1.0 (Crown)	1.0	10^−3^–10^−4^	1.0 × 10^−4^
Optode 2	49.0	49.0 (TCP)	1.0 (TFPBA)	1.0	10^−2^–10^−3^	1.0 × 10^−3^
Optode 3	49.0	49.0 (TCP)	1.0 (NPBA)	1.0	10^−1^–10^−3^	9.0 × 10^−4^
Optode 4	49.0	49.0 (NOPE)	1.0 (NPBA)	1.0	10^−1^–10^−2^	1.0 × 10^−2^

**Table 2:** ^1^H-NMR chemical shift of glucose, the ionophore NPBA, and their mixture in deuterated DMSO [39,40].

Glucose	NPBA	Mixture
4.405 (CH-OH_a_)	----	4.379
4.572, 4.588 (OH_d_)	----	4.397, 4.409
4.772 (OH_c_)	----	4.606
4.886 (OHe)	----	4.837, 4.850
4.899 (CH-OH_b_)		
6.151, 6.166 (OH_b_)	----	6.053, 6.065
----	8.615 (B-OH)	8.610

**Table 3:** Determination of glucose in different glucose-rich culture media.

Samples	Theoretical Value	Optode 3	Reference Method	Recovery
Synthetic culture media [3]	1.00 × 10^−3^ M	1.01 × 10^−3^ M	----	101.5%
	1.00 × 10^−4^ M	1.02 × 10^−4^ M	----	102.4%
Commercial culture Embryo media (GLOBAL^®^ TOTAL^®^ W/HEPES) [3]	----	2.04 × 10^−3^ M	2.01 × 10^−3^ M	101.5%
Serum from normal and diabetic patients [4]	----	155.8 mg/dL	144.0 mg/dL	108.2%
	----	382.2 mg/dL	370.0 mg/dL	103.3%

## Data Availability

Data is contained within the article or Supplementary Material.

## Supplementary Materials

The following supporting information can be downloaded at: https://www.mdpi.com/article/10.3390/s22197135/s1, Table S1. The effect of interfering materials at (10^−1^ glucose, 10^−1^ glucose+10^−2^ interfering species); Table S2. The effect of different interfering ions at different concentrations; Figure S1. ^1^H-NMR spectra of (**a**) glucose, (**b**) ionophore-NPBA, (**c**) their mixture (glucose-NPBA complex).
