# Supplementary material for: Non-Enzymatic Phenylboronic Acid-Based Optode Membrane for Glucose Monitoring in Serums of Diabetic Patients and in the Culture Medium of Human Embryos
Source: Sensors (Basel). 2022 Sep 21;22(19):7135. doi: 10.3390/s22197135 (PMC9571803; doi:10.3390/s22197135)
Supplement: Supplementary file 1 [file sensors-22-07135-s001.zip › sensors-1880509-supplementary.pdf]

sensors

*Article*

# Non-enzymatic Phenylboronic Acid-based Optode Membrane for Glucose Monitoring in Serums of Diabetic Patients and in the Culture Medium of Human Embryos

**Mohamed M. Taha [1,2], Mahmoud S. Rizk [1], Mohamed A. Zayed [1], Fatehy M. Abdel-Haleem [1,3] and Ahmed Barhoum [4,5,*]**

1 Chemistry Department, Faculty of Science, Cairo University, 12613 Giza, Egypt
2 Adam International Hospital, Giza, Egypt
3 Center for Hazards Mitigation, Environmental Studies and Research (CHMESR), Cairo University, Giza, Egypt
4 NanoStruc Research Group, Chemistry Department, Faculty of Science, Helwan University, 11795 Cairo, Egypt
5 National Centre for Sensor Research, Chemistry Department, Dublin City University, Dublin, Ireland
* Correspondence: ahmed.barhoum@dcu.ie

**Table S1.** The effect of interfering materials at $10^{-1}$ glucose, $10^{-1}$ glucose+$10^{-2}$ interfering species.

| Sample Solution | Absorbance (A) at λmax = 530 nm | Change in Absorbance (ΔA) Compared to Absorbance of the Glucose (0.578 a.u.) |
|---|---|---|
| Glucose | 0.578 | --- |
| Glucose + sodium pyruvate | 0.548 | −0.030 |
| Glucose + arginine | 0.624 | +0.046 |
| Glucose + glutamic acid | 0.574 | −0.004 |
| Glucose + tryptophan | 0.574 | −0.004 |
| Glucose + leucine | 0.597 | +0.019 |
| Glucose + sodium lactate | 0.609 | +0.031 |
| Glucose + phenylalanine | 0.599 | +0.021 |

**Table S2.** The effect of different interfering ions at different concentrations.

| Sample Solution | Absorbance (A) at λmax = 530 nm | Change in Absorbance (ΔA) Compared to Absorbance of the Glucose (0.576 a.u.) |
|---|---|---|
| $10^{-3}$ M glucose | 0.576 | --- |
| $10^{-3}$M glucose +$10^{-4}$M sodium pyruvate | 0.532 | −0.044 |
| $10^{-2}$ M glucose | 0.578 | --- |
| $10^{-2}$M glucose +$10^{-3}$M sodium pyruvate | 0.574 | −0.004 |
| $10^{-1}$ M glucose | 0.59 | --- |
| $10^{-1}$M glucose +$10^{-2}$M sodium pyruvate | 0.56 | −0.03 |
| $10^{-3}$ M glucose | 0.955 | --- |
| $10^{-3}$ M glucose + $10^{-4}$ M arginine | 0.996 | +0.041 |
| $10^{-2}$ M glucose | 0.952 | --- |
| $10^{-2}$ M glucose + $10^{-3}$ M arginine | 0.953 | +0.001 |
| $10^{-1}$ M glucose | 0.974 | --- |
| $10^{-1}$ M glucose + $10^{-2}$ M arginine | 1.02 | +0.046 |

| | | |
|---|---|---|
| $10^{-3}$ M glucose | 1.031 | --- |
| $10^{-3}$ M glucose + $10^{-4}$ M glutamic acid | 0.952 | −0.079 |
| $10^{-2}$ M glucose | 0.957 | --- |
| $10^{-2}$ M glucose + $10^{-3}$ M glutamic acid | 0.962 | +0.005 |
| $10^{-1}$ M glucose | 0.992 | --- |
| $10^{-1}$ M glucose + $10^{-2}$ M glutamic acid | 0.988 | −0.004 |
| $10^{-3}$ M glucose | 1.239 | --- |
| $10^{-3}$ M glucose + $10^{-4}$ M glutamic acid | 1.245 | +0.006 |
| $10^{-2}$ M glucose | 1.237 | --- |
| $10^{-2}$ M glucose + $10^{-3}$ M glutamic acid | 1.235 | −0.002 |
| $10^{-1}$ M glucose | 1.233 | --- |
| $10^{-1}$ M glucose + $10^{-2}$ M glutamic acid | 1.229 | −0.004 |
| $10^{-3}$ M glucose | 0.677 | --- |
| $10^{-3}$ M glucose + $10^{-4}$ M leucine | 0.753 | +0.076 |
| $10^{-2}$ M glucose | 0.738 | --- |
| $10^{-2}$ M glucose + $10^{-3}$ M leucine | 0.812 | +0.074 |
| $10^{-1}$ M glucose | 0.716 | --- |
| $10^{-1}$ M glucose + $10^{-2}$ M leucine | 0.735 | +0.019 |
| $10^{-3}$ M glucose | 0.554 | --- |
| $10^{-3}$ M glucose + $10^{-4}$ M lactate | 0.601 | +0.047 |
| $10^{-2}$ M glucose | 0.592 | --- |
| $10^{-2}$ M glucose + $10^{-3}$ M lactate | 0.63 | +0.038 |
| $10^{-1}$ M glucose | 0.656 | --- |
| $10^{-1}$ M glucose + $10^{-2}$ M lactate | 0.687 | +0.031 |
| $10^{-3}$ M glucose | 1.083 | --- |
| $10^{-3}$ M glucose + $10^{-4}$ M phenylalanine | 1.095 | +0.012 |
| $10^{-2}$ M glucose | 1.107 | --- |
| $10^{-2}$ M glucose + $10^{-3}$ M phenylalanine | 1.142 | +0.035 |
| $10^{-1}$ M glucose | 1.07 | --- |
| $10^{-1}$ M glucose + $10^{-2}$ M phenylalanine | 1.091 | +0.021 |

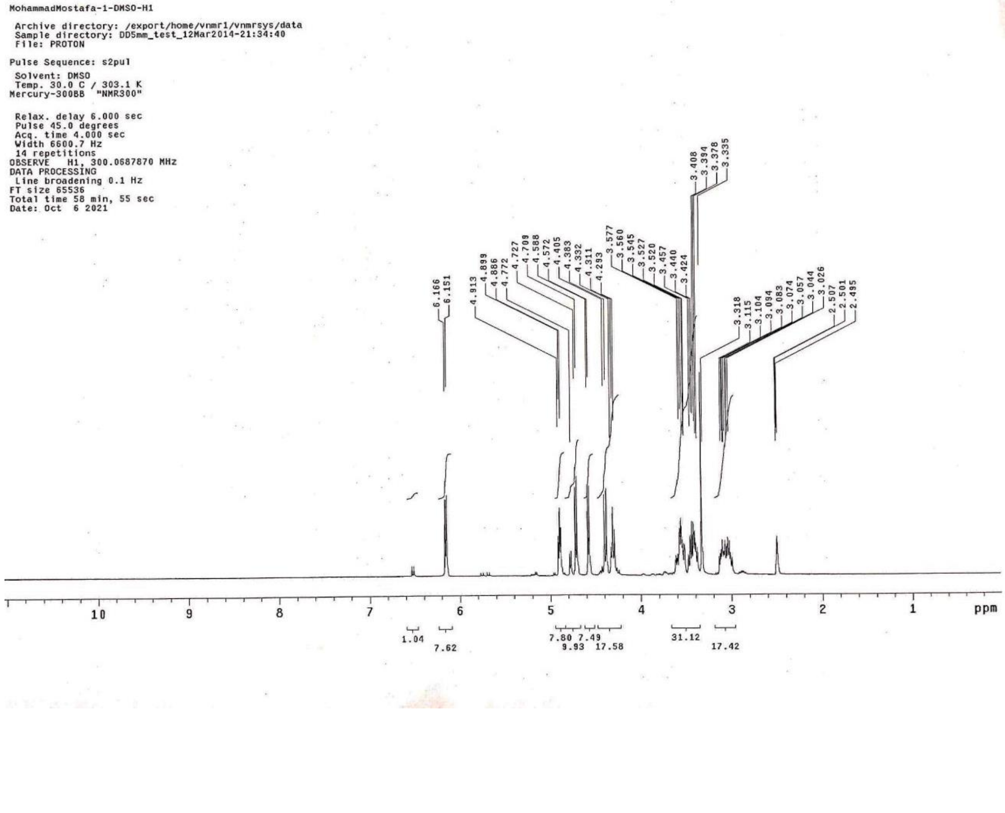

(**a**)

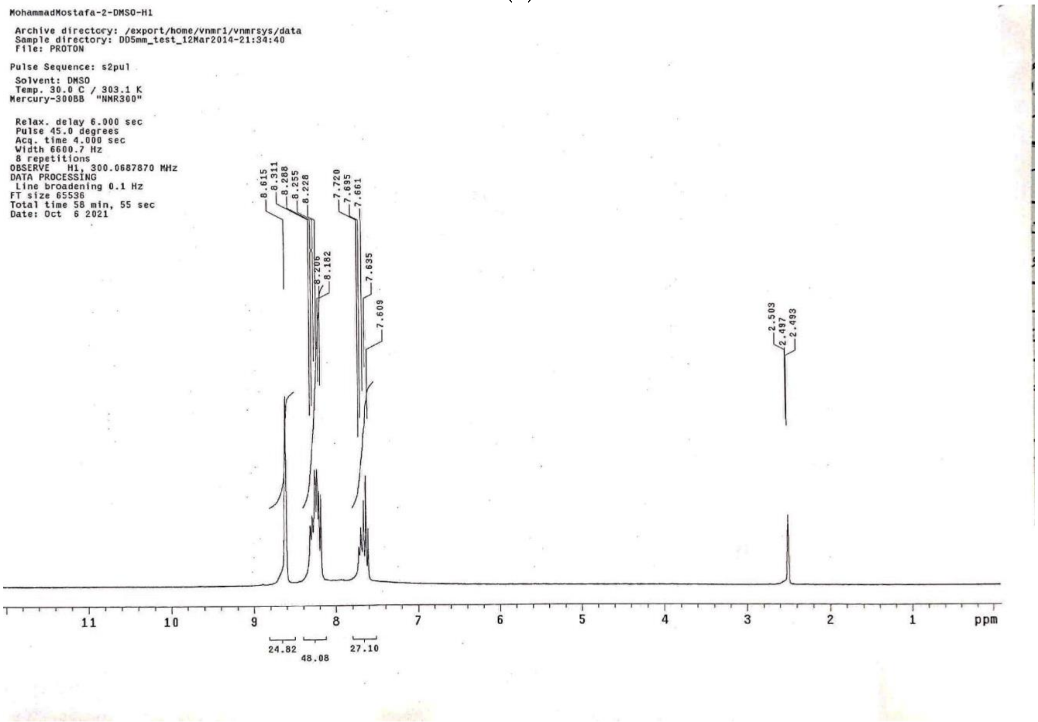


(**b**)

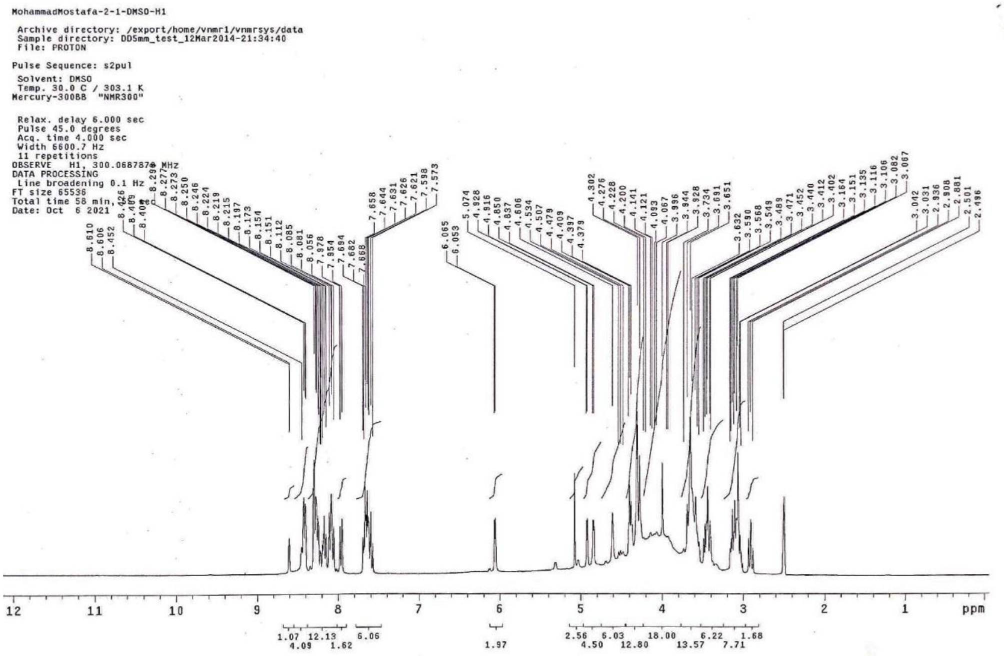


(**c**)

**Figure S1.** $^1$H-NMR spectra of (**a**) glucose, (**b**) ionophore-NPBA, and (**c**) their mixture (glucose–NPBA complex).
